# Transcriptome Analysis of Potential Genes Involved in Innate Immunity in Mudflat Crab (*Helice tientsinensis*)

**DOI:** 10.3390/ani15192855

**Published:** 2025-09-30

**Authors:** Lulu Chen, Ming Wang, Mengdi Zhou, Youkun Fang, Tingting Ji, Ruyang Xia, Menglu Bai, Zhengfei Wang, Jiafei Shen

**Affiliations:** Jiangsu Key Laboratory for Bioresources of Saline Soils, Jiangsu Synthetic Innovation Center for Coastal Bio-Agriculture, Jiangsu Provincial Key Laboratory of Coastal Wetland Bioresources and Environmental Protection, School of Wetlands, Yancheng Teachers University, No. 2, South Hope Avenue, Yancheng 224051, China; chenll@yctu.edu.cn (L.C.); wming31@outlook.com (M.W.); zhoumd1129@outlook.com (M.Z.); 15578748197@163.com (Y.F.); jitingting666@outlook.com (T.J.); xiaruyang7@gmail.com (R.X.); ml17322236@outlook.com (M.B.)

**Keywords:** infection, mudflat crab, qRT-PCR, RNA sequencing, *Vibrio parahaemolyticus*

## Abstract

**Simple Summary:**

*Vibrio* species are widely recognized as highly pathogenic bacteria in aquaculture, causing extensive mortality among cultured organisms and resulting in substantial economic losses. *H. tientsinensis*, a dominant crustacean species in the Yellow River Delta, has been the subject of limited investigation regarding its susceptibility to *Vibrio* infection. In this study, we examined structural alterations and differential gene expression in the gill and hepatopancreatic tissues of *H. tientsinensis* following infection with Vibrio parahaemolyticus, and further explored the associated metabolic and immune responses. The findings provide valuable insights into the defense mechanisms of crustaceans against Vibrio pathogens and offer a scientific foundation for aquaculture management and targeted disease prevention strategies.

**Abstract:**

The mudflat crab (*H. tientsinensis*) is a dominant species in coastal tidal flat areas, primarily inhabiting the high tide region of the intertidal zone, and possesses significant ecological and economic value. *Vibrio* species are one of the main bacterial pathogens responsible for diseases in marine organisms, and they are widely distributed in seawater and estuarine environments. However, the immune mechanisms employed by *H. tientsinensis* in response to *Vibrio* infections remain unclear. This study aims to investigate the physiological and immune mechanisms by analyzing the structural changes and differential gene expression in the gill and hepatopancreas following *Vibrio parahaemolyticus* infection. The results indicate that *V. parahaemolyticus* infection causes cellular damage, with structural alterations observed in the gills (epithelial cell edema in the gill filaments, and aneurysm formation) and the hepatopancreas (changes in lumen size, nuclear condensation, and modifications in connective tissue morphology). Transcriptome analysis revealed 9766 differentially expressed genes (DEGs) in the gills of the experimental group, with 4687 upregulated and 5079 downregulated genes. These DEGs are primarily involved in different ribosomal subunits. In the hepatopancreas, 1594 DEGs were identified, with 834 upregulated and 760 downregulated. These DEGs are predominantly associated with energy-coupled proton transmembrane transport, electron transport-coupled proton transport, and lipid transporter activity. *H. tientsinensis* gene annotation and KEGG enrichment analysis revealed that chemical carcinogens DNA adducts, amino acid metabolism, and some immune pathways play key roles in the ability of *H. tientsinensis* to defend against *V. parahaemolyticus* infection. The findings of this study contribute to a deeper understanding of the immune mechanisms of *H. tientsinensis* against *V. parahaemolyticus* infection and provide new insights for aquaculture management.

## 1. Introduction

*H. tientsinensis* (Brachyura: Grapsoidea: Varunidae) is widely distributed along the coast of the West Pacific, primarily inhabiting intertidal mudflats, swamps, salt marshes, and estuarine areas, and it is a key component of the Yellow River Delta ecosystem, possessing significant ecological and economic value [1,2]. *H. tientsinensis* plays a crucial role in ecological restoration and maintaining ecological balance. *H. tientsinensis* mainly feeds on the *Phragmites australis*, *Spartina alterniflora*, and *Suaeda salsa* in the mudflat [1,3]. The invasion of *S. alterniflora* has increased the amount of plant organic debris, caused an increase in the number of macrobenthos feeding on plant organic debris, reduced the biomass of benthic microalgae, affected the survival of small benthos feeding on microalgae, and destroyed the biodiversity of the mudflat [4,5]. *H. tientsinensis* helps suppress the growth of the invasive species *S. alterniflora* by consuming its seedlings [6,7]. Additionally, *H. tientsinensis* continuously scrapes the surface of the soil in salt marsh habitats, removing *S. alterniflora* detritus. This activity significantly reduces the organic matter content in the soil, limits the density of macrobenthos, and prevents *S. alterniflora* from occupying the ecological niches of other species, thereby contributing to the stability of the ecosystem [8,9,10]. As an integral part of the coastal ecosystem, *H. tientsinensis* not only consumes plants growing in the intertidal zone, such as *Suaeda salsa*, *Salicornia europaea*, *Phragmites australis*, and *S. alterniflora*, as well as microorganisms in seawater, but it also serves as an important food source for various migratory bird species, including gulls, curlews, cranes, and carnivorous fish in the coastal wetlands, at their stopover or wintering sites. *H. tientsinensis* is known for its delicious meat, which is highly favored by coastal populations and holds considerable economic value [11].

Crustaceans lack the acquired immune system found in vertebrates and primarily rely on innate immunity to defend against various pathogenic microorganisms in their environment [12,13]. Bacteria are the most widespread and abundant pathogens causing diseases in marine shrimp and crab populations, with species of *Vibrio* serving as the primary bacterial pathogens responsible for infections in crustaceans [14,15]. Pathogenic vibrios, such as *V. parahaemolyticus*, *Vibrio harveyi*, and *Vibrio alginolyticus*, are significant contributors to these infections [16,17]. *V. parahaemolyticus*, a naturally occurring halophilic Gram-negative bacterium, is widely distributed in marine and estuarine environments and poses a serious threat to economically important shrimp and crab species. Infections caused by *V. parahaemolyticus* in *Litopenaeus vannamei* include hepatopancreatic damage and are characterized by clinical symptoms such as hepatopancreatic damage, lesions in the stratum corneum or epithelial tissues, disruption of structural barriers, softening of the shell, intestinal atrophy or discontinuity, loss of internal contents, and pale discoloration, often resulting in mortality rates as high as 100% [18,19]. Previous studies have demonstrated that *V. parahaemolyticus* accelerates energy consumption in the hepatopancreas [20,21], causing significant damage to the organ, leading to intestinal atrophy, and resulting in the permeation of endotoxins from bacterial cell walls into tissues [22]. Ultimately, this results in hepatopancreatic failure and necrosis, as evidenced by pathological changes in *Eriocheir sinensis* [20]. Additionally, *V. parahaemolyticus* impedes the growth of *Portunus trituberculatus*, and causes difficulties during the molting process [23]. Moreover, the synthase activity regulating the amino acid metabolic pathway in *Scylla paramamosain* is inhibited following *V. parahaemolyticus* infection [24], leading to significant economic losses in coastal aquaculture and fisheries [25,26].

Transcriptome analysis is a highly effective method for conducting systematic studies of gene function and is also a valuable approach for exploring gene expression levels, providing a comprehensive overview of complex biosynthesis processes [27]. While studies on the genomics of *V. parahaemolyticus* in common economic crustaceans have been reported, the immune response mechanisms of *H. tientsinensis* against *V. parahaemolyticus* remain unclear. This study aims to investigate the tissue morphology and differential gene expression in the gills and hepatopancreas of *H. tientsinensis* following *V. parahaemolyticus* infection, through pathological tissue sections and transcriptome analysis, to identify key genes associated with *V. parahaemolyticus* infection in *H. tientsinensis*. Investigating the changes in metabolism and immune responses of *V. parahaemolyticus* infection in *H. tientsinensis* will contribute to a deeper understanding of the innate immune system. This research will enrich the knowledge of crustacean mechanisms against *Vibrio* infections and provide theoretical guidance for scientific aquaculture practices.

## 2. Materials and Methods

### 2.1. Experimental Samples

*H. tientsinensis* (Carapace length: 2.5–2.9 cm, Carapace width: 3.2–3.7 cm, body weight: 18.1–25.0 g) male samples (*n* = 20) were collected from the coastal tidal flats (33.27578964° N, 120.79251467° E) of Dafeng Port in Yancheng, Jiangsu Province, China, along with soil and water samples from the surrounding area. The samples were temporarily maintained in a simulated tidal flat environment at 25 °C in the laboratory. During the maintenance period, water and food were supplemented as needed, and deceased samples were promptly removed.

### 2.2. V. parahaemolyticus Infection and Sample Collection

The *V. parahaemolyticus* strain (ATCC 17802) used in this study was obtained from the China General Microbiological Culture Collection Center (CGMCC) and stored in a −80 °C ultra-low temperature freezer (Thermo Fisher Scientific, Waltham, MA, USA). *V. parahaemolyticus* was revived by culturing in 3% NaCl nutrient agar liquid medium (10 g peptone, 5 g beef extract, 30 g NaCl dissolved in 1 L ddH_2_O, pH = 7.0) until an OD_600_ reached 0.6. Then, the bacterial cells were centrifuged at 4000 rpm for 10 min and suspended in phosphate-buffered saline (PBS) for collection. Mature crabs were randomly divided into two groups: the control group (PBS group) and the experimental group (*V. parahaemolyticus* infection group, VP group). Each group consisted of three biological replicates. In the experimental group, 50 μL of *V. parahaemolyticus* (2.76 × 10^7^ CFU/mL) was injected into the third walking leg joint membrane of each crab. The control group received the same volume of PBS. 48 h after injection, the gill and hepatopancreas tissues were collected for dissection. One portion was fixed in 4% paraformaldehyde (Biosharp, Chengdu, Sichuan, China) at 4 °C for subsequent histopathological analysis, while the other portion was snap-frozen in liquid nitrogen and stored at −80 °C for RNA extraction and transcriptome sequencing.

### 2.3. Histopathological Investigation

The fixed tissues of *H. tientsinensis* were cut into appropriately sized pieces (approximately 1 cm) using a surgical blade in the fume hood (Fuxia, Shaoxin, Zhejiang, China). The tissues were then subjected to a series of graded ethanol dehydration steps followed by paraffin embedding [28]. The embedded paraffin blocks were sectioned into 6 μm-thick sections using a microtome (Leica, Wetzlar, Germany). The paraffin sections were stained with Hematoxylin and Eosin (H&E), and the morphological features and pathological changes in the gill and hepatopancreas tissues were observed and photographed under an optical microscope (Leica, Wetzlar, Germany).

### 2.4. RNA Extraction, cDNA Library Construction, and Sequencing

Total RNA from the gill and hepatopancreas tissues was extracted using the RNAprep Pure Tissue Kit (Tiangen, Beijing, China) according to the manufacturer’s procedure. The RNA concentration and purity were measured using a Nanodrop 2000 spectrophotometer (Thermo Fisher Scientific, Waltham, MA, USA), and RNA integrity was verified by 1.5% agarose gel electrophoresis. The statistical power for our RNA-seq data was calculated using the RNA-Seq Power Calculator [29]. Three biological replicates were employed to achieve the required statistical power. cDNA libraries were generated using the Illumina Truseq™ RNA Sample Prep Kit (Illumina, San Diego, CA, USA). Subsequently, all sample libraries were sequenced on the Illumina HiSeq/MiSeq platform (Illumina, San Diego, CA, USA), with sequencing conducted by Majorbio Bio-Pharm Technology Co., Ltd. (Shanghai, China).

### 2.5. Transcriptome Assembly and Functional Annotation

After high-quality RNA-seq sequencing data were obtained, reads containing poly-N reads (>10% reads) or low-quality reads (<20 bp reads, q-value < 20) were removed using SeqPrep (version 0.1) (https://github.com/jstjohn/SeqPrep, accessed on 5 May 2023) and Sickle (version 1.33) (https://github.com/najoshi/sickle, accessed on 10 May 2023) to obtain the clean reads. De novo assembly of all clean data was performed using Trinity software (version 2.15.2) (https://github.com/trinityrnaseq/trinityrnaseq/wiki, accessed on 3 June 2023). The assembly results of the initial reads were then evaluated, and redundant reads were removed using TransRate software (version 1.0.3) (http://hibberdlab.com/transrate/, accessed on 10 June 2023) and CD-HIT software (version 4.6.2) (https://github.com/weizhongli/cdhit/archive/V4.6.2.tar.gz, accessed on 12 June 2023). The transcriptome was evaluated using BUSCO software (version 6.0.0) (Benchmarking Universal SingleCopy Orthologs, http://busco.ezlab.org, accessed on 20 June 2023) to ensure the completeness of the assembly. All transcripts obtained from RNA-seq sequencing were compared against six major databases: non-redundant protein sequence (NR, https://www.ncbi.nlm.nih.gov/public/, accessed on 4 July 2023); Swiss-Prot (https://ucrania.imd.ufrn.br/~pitagoras/protein_dimension_db/release_1/uniprot_sorted.fasta.gz, accessed on 5 July 2023); Pfam (https://pfam.sanger.ac.uk/, accessed on 6 July 2023); EggCOG (http://eggnogdb.embl.de/#/app/home, accessed on 10 July 2023); Gene Ontology database (GO, https://www.geneontology.org, accessed on 19 July 2023); and Kyoto Encyclopedia of Genes and Genomes (KEGG, https://www.genome.jp/kegg/, accessed on 5 August 2023). BLAST+ (https://ftp.ncbi.nlm.nih.gov/blast/executables/blast+/LATEST/, accessed on 15 September 2023) was used for obtaining the annotation information from each database, and statistics were compiled for each database’s annotation results.

### 2.6. Analysis of DEGs

The expression levels of genes/transcripts were analyzed using RSEM (RNA-Seq by Expectation-Maximization) software (version 1.3.3) (https://github.com/deweylab/RSEM, accessed on 5 April 2024) to define the differential expression between samples. Meanwhile, the functional annotation of transcripts was integrated to uncover the regulatory mechanisms of genes. TPM (Transcript per million) data for transcripts and genes were obtained by correcting gene length variations across samples, caused by differential transcript usage, using the tximport 1.10.0 R package and the “length Scaled TPM” tool. Additionally, the corrected read count data for each gene was used to evaluate gene expression levels in terms of fragments per kilobase of transcript per million fragments mapped (FPKM). The corrected read count data for each gene were then imported into EdgeR (version 4.7.5) (http://bioconductor.org/packages/stats/bioc/edgeR/, accessed on 10 September 2024) for statistical comparison between experimental and control groups. DEGs were considered when the false discovery rate (FDR) was ≤0.05 and the FPKM was ≥1. GO functional and KEGG pathway enrichment analyses were performed to map all DEGs to different categories, aiming to understand the functions and biological pathways involved. GO functional enrichment analysis was conducted using Goatools (version 1.4.12) (https://github.com/tanghaibao/Goatools, accessed on 5 October 2024), with a p-value threshold of 0.05. KEGG pathway enrichment for candidate genes was analyzed using KOBAS (version 3.0) (http://bioinfo.org/kobas/, accessed on 15 October 2024), and statistical significance was considered when FDR ≤ 0.05.

### 2.7. qRT-PCR Validation

To validate the RNA-seq results by qRT-PCR, six DEGs were randomly selected from the PBS and VP groups, including peroxisomal catalase-like (*PXCAT*), acid phosphatase type 7-like (*ACP7*), anti-lipopolysaccharide factor-like (*ALF*), inhibitor of apoptosis protein 1 (*IAP1*), caspase-1-like (*CASP1*), and alkaline phosphatase-like (*AKP*). Glyceraldehyde-3-phosphate dehydrogenase (*GAPDH*) was employed as the reference gene. The same RNA samples used for RNA-Seq library construction were reverse transcribed into cDNA libraries. All qPCR primers were designed using Primer Premier 5.0 software and listed in Table S1. The qRT-PCR reactions were performed in a 20 µL reaction volume, consisting of 10 µL of 2 × FastReal qPCR PreMix (TIANGEN, Beijing, China), 0.6 µL of forward and reverse primers, 2 µL of 50 × cDNA, 0.4 µL of ROX, and 6.4 µL of RNase-free ddH_2_O. The reactions were then run on an ABI QuantStudio 3 quantitative PCR instrument (Applied Biosystems, Foster City, CA, USA). The thermal cycling conditions were set as follows: 95 °C for 2 min, 45 cycles at 95 °C for 5 s, 55 °C for 10 s, and 72 °C for 30 s. The relative expression levels of different genes were calculated according to the 2^−ΔΔCT^ method [30]. Three biological replicates were included for each gene.

## 3. Results

### 3.1. Histopathological Evaluation

**Figure 1:**
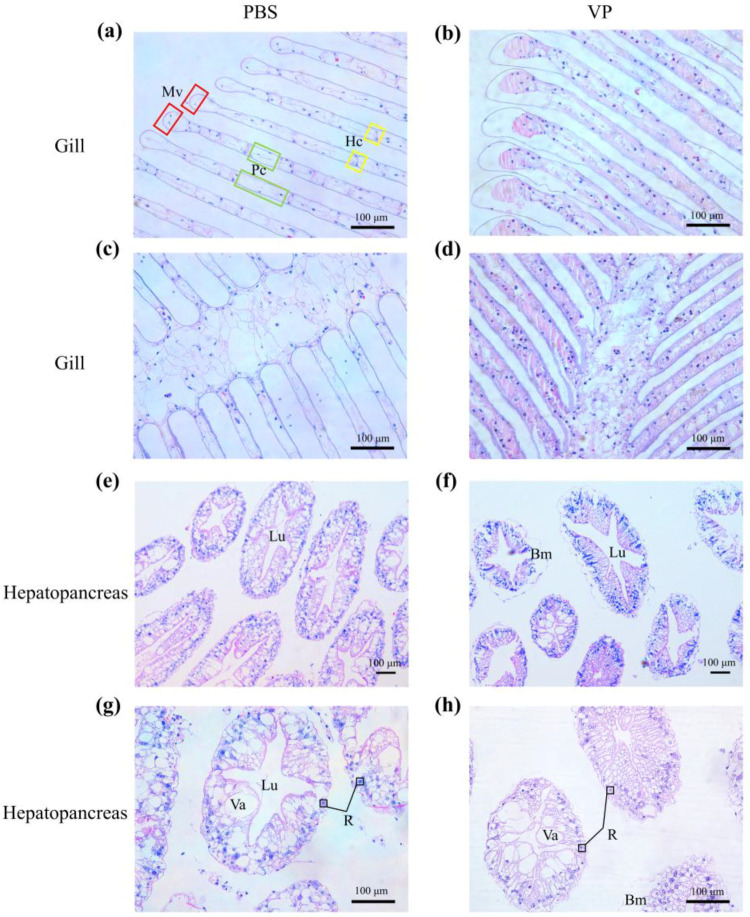
Histological sections of *H. tientsinensis* 48 h after treatment with phosphate-buffered saline (PBS group) or *V. parahaemolyticus* (VP group). (**a**) The morphology of the gill filaments 48 h after PBS injection. (**b**) The morphology of the gill filaments 48 h after VP injection. (**c**) The morphology of the gill axis 48 h after PBS injection. (**d**) The morphology of the gill axis 48 h after VP injection. (**e**,**g**) The morphology of the hepatopancreas 48 h after PBS injection. (**f**,**h**) The morphology of the hepatopancreas 48 h after VP injection. Mv, Marginal vessel; Hc, Blood cell; Pc, Pillar cell; Bm, Basement membrane; Lu, Lumen; Va, Vacuolization; R, Absorption cell. The red box represents Mv, the green box represents Pc, and the yellow box represents Hc. Scale bar, 100 μm.

After 48 h of *V. parahaemolyticus* infection, the gills of *H. tientsinensis* displayed distinct changes. In the PBS control group, the gill filaments were neatly arranged, with no signs of gill cavity dilation or rupture. The blood cells of the gill filaments were separated by pillar cells, and the marginal vessel was obvious (Figure 1a,c). In the VP experimental group, the gill cavity was dilated, gill filaments were ruptured, the structure of the blood cells and the pillar cells became blurred, the blood cells were aggregated in the gill lobules, the number of blood cells was increased, and the end was enlarged (Figure 1b,d). In the hepatopancreas tissue, the lumen morphology of the hepatopancreas tubules in the PBS control group was normal, maintaining a stellate shape, with well-defined connective tissue and evenly distributed nuclei located near the outer edge of the hepatopancreas tubules (Figure 1e,g). In the hepatopancreas tubules of the VP experimental group, nuclei collapsed, and the detachment of the basement membrane occurred. The star-shaped structure of the hepatopancreas expanded, the folds contracted, and in some cases, the lumen space of the hepatopancreas tubules increased. Epithelial cells atrophy, disappearance of the stellate shape, as well as the enlargement and increase in vacuoles in hepatopancreas tubules were seen (Figure 1f,h).

### 3.2. De Novo Assembly and Annotation of Unigenes

The total number of raw reads from both the control and experimental groups was confirmed through RNA sequencing (Table S2). After the removal of poly-N sequences and low-quality reads, the average percentage of clean reads obtained from each sample was 98.47%. Mapping to the reference genome, the average mapping ratio at the transcript level for each sample was 83.39%. Subsequently, de novo assembly of the clean reads was performed using Trinity software, resulting in a total of 95,878 reads, of which 13,014 reads exceeded 1500 bp in length (Table S3). PCA analysis was conducted on transcriptome sequencing data from gill and hepatopancreatic tissues. The four groups (PBS group of gills, VP group of gills, PBS group of hepatopancreas, VP group of hepatopancreas) exhibited a completely separated quadrant distribution (Figure S1). A total of 39,743 non-redundant unigenes were annotated in public databases. Of these, 20,962 were assigned to the GO database, 19,668 to the KEGG, 24,074 to eggNOG, 36,689 to the NR protein database, 19,375 to Swiss-Prot, and 20,793 to Pfam (Table 1).

In GO database, those unigenes were classified into three categories: biological process (BP), cellular component (CC), and molecular function (MF). The most prevalent terms in the BP category were cellular process (8266 unigenes) and metabolic process (7817 unigenes). In the CC category, cell part (8315 unigenes) and membrane (6031 unigenes) were the dominant terms. For the MF category, binding (10,675 unigenes) and catalytic activity (11,024 unigenes) were the most abundant subcategories (Figure S2a). A total of 19,668 unigenes, primarily involved in seven categories including Metabolism, Genetic Information Processing, Environmental Information Processing, Cellular Processes, Organismal Systems, Human diseases, and Drug Development, were mapped to 46 pathways in the KEGG database, with the three most enriched pathways being Infectious disease: Viral, Translation, and Signal transduction (Figure S2b).

### 3.3. Classification and Analysis of DEGs

**Figure 2:**
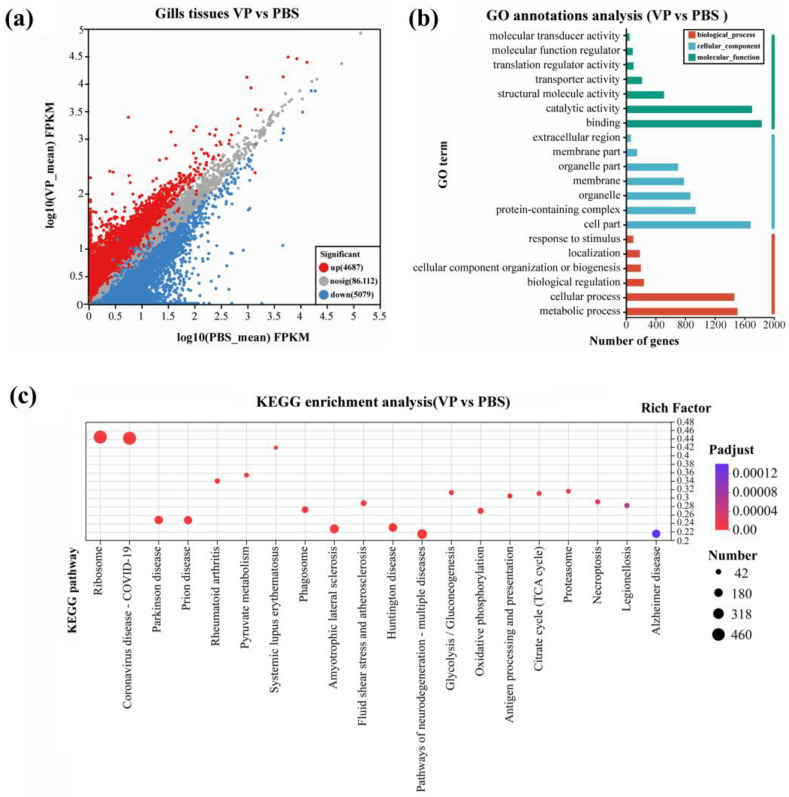
Functional annotation and enrichment of DEGs in gills at 48 h after PBS or VP treatments. (**a**) Scatter of the DEGs volcano on the gill. The abbreviation VP denotes the *Vibrio parahaemolyticus* infection group, while PBS refers to the phosphate-buffered saline control group. (**b**) Functional annotation and classification of DEGs in the gills. (**c**) Scatterplot of KEGG pathway enrichment for gills. The Functional annotation and classification of DEGs display the top twenty annotations for the feature.

**Figure 3:**
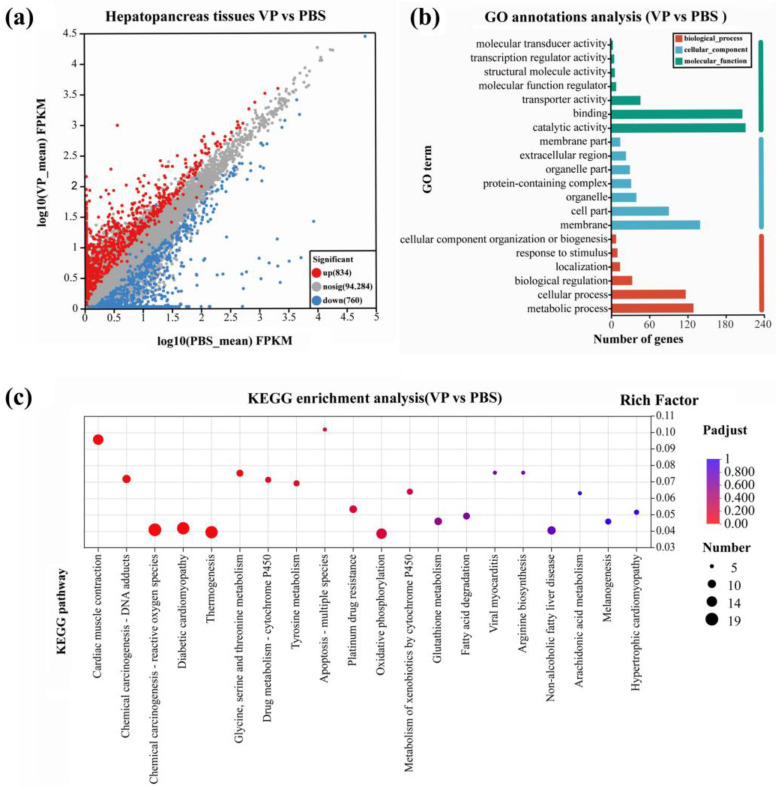
Functional annotation and enrichment of DEGs in hepatopancreas at 48 h after PBS or VP treatments. (**a**) DEGs scatter map of the hepatopancreas. The abbreviation VP denotes the *Vibrio parahaemolyticus* infection group, while PBS refers to the phosphate-buffered saline control group. (**b**) Functional annotation and classification of DEGs in hepatopancreas. (**c**) Scatterplot of KEGG pathway enrichment for hepatopancreas. The Functional annotation and classification of DEGs display the top twenty annotations for the feature.

To further analyze and characterize the DEGs, a threshold was applied to select DEGs with a significance level of less than 0.05 (P*adjust* < 0.05) and gene expression changes of at least 2-fold (|log2FC| ≥ 1). After the injection of *V. parahaemolyticus* into the *H. tientsinensis*, 9766 DEGs were detected in the gills (4687 upregulated and 5079 downregulated) (Figure 2a; Table 2), while 1594 DEGs were identified in the hepatopancreas (834 upregulated and 760 downregulated) (Figure 3a, Table 3). Among these DEGs, 276 were significantly upregulated or downregulated in both the gill and hepatopancreas tissues.

### 3.4. GO and KEGG Analysis of DEGs

GO annotation analysis revealed that the 9766 DEGs in the gill were classified into three categories, with a total of 45 functional groups: 19 groups in biological process (predominantly associated with cellular and metabolic processes), 12 in cellular component (primarily linked to cell parts, organelles, and protein-containing complex), and 14 in molecular function (chiefly involved in binding, catalytic activity, and transporter activity) (Figure 2b). The 1594 unigenes identified in the hepatopancreas were classified into three main categories across 39 functional groups. These categories included biological processes (16 functional groups), cellular components (12 functional groups), and molecular functions (11 functional groups) (Figure 3b). Analysis of biological processes identified that a substantial proportion of DEGs were predominantly involved in cellular, metabolic, and signaling processes. Investigation of cellular components revealed that over fifty percent of the DEGs were associated with cell parts, membranes, and components of membrane structures. In terms of molecular functions, DEGs were chiefly linked to binding, catalytic activity, and transporter activity.

The KEGG enrichment analysis revealed significant enrichment of pathways related to ribosome, coronavirus disease COVID-19, and systemic lupus erythematosus in the gill (Figure 2c). In contrast, the DEGs in the hepatopancreas were significantly enriched in pathways associated with cardiac muscle contraction, chemical carcinogenesis DNA adducts, and glycine, serine, and threonine metabolism (Figure 3c).

### 3.5. Validation of RNA-Seq Data by qRT-PCR

**Figure 4:**
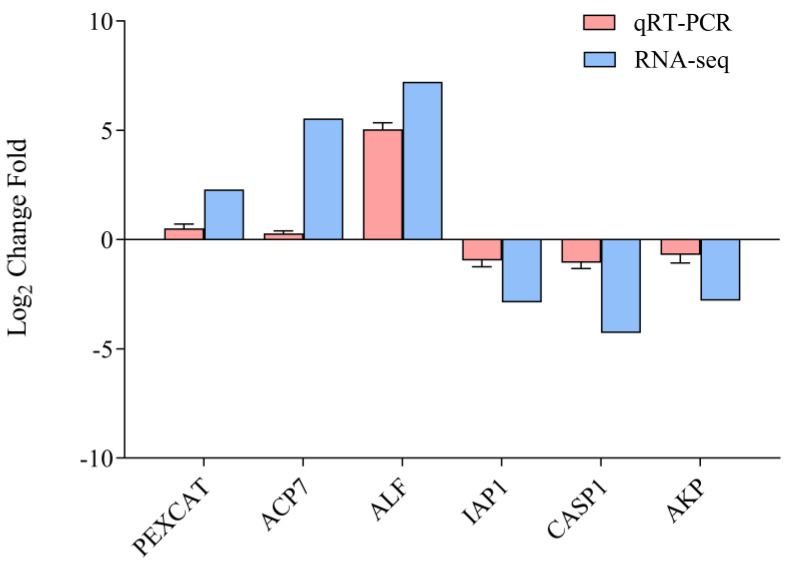
Validation of transcriptome data using qRT−PCR. DEG analysis between *Vibrio parahaemolyticus* (VP) and phosphate-buffered saline (PBS) groups in the gills and hepatopancreas of *H. tientsinensis*. *PXCAT*, peroxisomal catalase-like; *ACP7*, acid phosphatase type 7−like; *ALF*, anti-lipopolysaccharide factor−like; *IAP1*, inhibitor of apoptosis protein 1; *CASP1*, caspase−1−like; *AKP*, alkaline phosphatase−like. In the verification experiment, *PXCAT*, *ACP7*, and *ALF* were identified as DEGs in the gills, while *IAP1*, *CASP1*, and *AKP* were DEGs in the hepatopancreas. *GAPDH* was used as the reference gene. Data are presented as mean ± S.D. from three biological replicates (*n* = 3).

Six genes from the RNA-seq data were selected for qRT-PCR analysis. The qRT-PCR expression patterns of these six genes (*PXCAT*, *ACP7*, *ALF*, *IAP1*, *CASP1*, and *AKP*) showed consistent agreement with RNA-seq results (Figure 4). This indicates that the expression trends of the genes measured through RNAseq were accurate and reliable.

## 4. Discussion

The gill, serving as the respiratory organ of crustaceans, acts as a direct interface between the organism and its environment. When crustaceans are exposed to pathogens and toxins, the gill, as the first line of defense, directly encounters the pathogens and activates immune-related metabolic pathways [31]. Additionally, the gill plays a crucial role in respiration, osmoregulation, excretion of nitrogenous wastes, and maintenance of acid-base balance, making it a vital organ to consider in toxicological analyses [32,33], the hepatopancreas of crustaceans, analogous to the liver, pancreas, and intestine of vertebrates, plays an essential role in metabolism, nutrient absorption, and immune function [34]. As the primary detoxification organ for exogenous substances, the hepatopancreas also plays a significant role in oxidative stress [35].

This study found that after *V. parahaemolyticus* infection for 48 h, significant damage was observed in the gill and hepatopancreas tissues of *H*. *tientsinensis*. According to the DEGs analysis, the expression of Aspartate aminotransferase (*AST*) was significantly upregulated in the hepatopancreas of the experimental group of *H*. *tientsinensis* following infection with *V. parahaemolyticus*, while both *AST* gene and Alanine aminotransferase (*ALT*) *gene* expressions were significantly increased in the gill tissue. These findings are consistent with results from *E. sinensis* infected by *V. parahaemolyticus*, where changes in the gene expression levels of *ALT* and *AST* were also reported. ALT and AST proteins are transferase enzymes [36], and the significant increase in their activities indicates that tissue damage occurred in the crab. DEG enrichment analysis revealed that significantly altered genes were associated with immune response, oxidative stress, and apoptosis. Several GO terms, including transporter activity (GO:0005215), immune system process (GO:0002376), and biological regulation (GO:0065007), were linked to immune responses. Additionally, the KEGG enrichment analysis highlighted significant enrichment of DEGs in immune and apoptosis-related pathways, such as phagosome (K06563) and lysosome (K19363).

### 4.1. Immune-Related Genes

After 48 h of *V. alginolyticus* infection in *H. tientsinensis*, the expression level of Lysozyme gene (*LZM*) in the gill was significantly upregulated, while a similar molecule of *LZM* in the hepatopancreas was found to be significantly downregulated. LZM is an alkaline protein capable of lysing bacterial cell walls and exhibiting a strong inhibitory effect on *V. alginolyticus* and *V. parahaemolyticus* [37,38]. LZM is also an antimicrobial peptide (AMP) that is capable of killing and clearing invading bacteria [39,40]. The downregulation of *LZM* gene expression in the hepatopancreas was also observed 12 h after *V. parahaemolyticus* infection in *E. sinensis* [20]. Based on these observations, it is speculated that the immune-related mechanisms in the hepatopancreas respond rapidly to pathogen invasion, and LZM activity is suppressed in the later stages of infection. In contrast, the increase in LZM activity in the gill of *H. tientsinensis* suggests that the immune defense mechanisms in crustaceans may be activated during the early contact phase with pathogens and continue to respond to immune challenges in order to resist the invasion of exogenous substances.

In addition, another antimicrobial peptide, ALF, which plays a crucial role in the innate immunity of crustaceans [41,42], was found to be significantly upregulated in the gill of *E. sinensis* following microbial infection. In the present study, two types of *AMP* genes (*LZM* and *ALF*) were upregulated after *V. parahaemolyticus* infection, suggesting that both LZM and ALF may play key roles in the immune defense against microbial infection in *H. tientsinensis*.

Noteworthy, the expression level of acid phosphatase type (*ACP*) gene, which encoded an enzyme associated with non-specific immune response, was significantly upregulated in the gill of *H. tientsinensis* in the experimental group. In contrast, the expression levels of the *ACP* and *AKP* gene were significantly reduced in the hepatopancreas of the experimental group. ACP plays an essential role in various important immune defense activities [43] and is involved in the transfer and metabolism of phosphate groups [44]. In crustaceans, AKP is also an important metabolic-regulatory enzyme, working in conjunction with other hydrolases to form a crucial detoxification system [45]. Both ACP and AKP regulate cellular metabolism within the immune defense system [46,47]. Following *V. alginolyticus* infection in *L. vannamei*, the expression of the *AKP* gene in the hepatopancreas was found to be significantly lower than in the control group [48]. In *E. sinensis*, after infection with *V. parahaemolyticus*, the expression level of *ACP* in the hepatopancreas was significantly higher in the experimental group compared to the control group [20]. Based on these findings, it is hypothesized that ACP in the gill of *H. tientsinensis* is involved in immune regulation, accelerating the metabolism and transport of immune-related substances. However, the activity of metabolic-regulatory enzymes and the function of the detoxification system in the hepatopancreas are somewhat inhibited following pathogen infection.

Following *V. parahaemolyticus* infection in *H. tientsinensis*, the expression levels of Scavenger receptors (*SR*) gene and C-type lectin domain family 6 member A-like (*CTL*) gene were significantly upregulated in the gill of the experimental group. SR is a typical subfamily of pattern recognition receptors (PRRs) [49,50,51], while CTL is a broad family of PRRs [52]. High expression of *SR* was observed in the hepatopancreas and gill of *E. sinensis*, where it binds to both Gram-negative and Gram-positive bacteria, enhancing AMP production and phagocytosis during bacterial invasion [53,54]. Following an attack by Gram-negative bacteria, *E. sinensis CTLs* are significantly upregulated [55]. These findings suggest that SR and CTL in the gill of *H. tientsinensis* are involved in immune recognition, antimicrobial activity, microbial binding, and immune regulation, playing a critical role in innate immunity. After a pathogen attack, PRRs in the gill of *H. tientsinensis* activate corresponding immune pathways, inducing the upregulation of *AMP* expression and contributing to the host’s innate immune defense.

The DEG analysis revealed that the expression level of Lipopolysaccharide-induced TNF-α factor (*LITAF*) was significantly upregulated in the gill of *H. tientsinensis*. *LITAF* is an immune-related gene induced by lipopolysaccharide (LPS) and plays a critical role in immune regulation in response to *Vibrio* species and viruses [56]. *LITAF* regulates inflammatory and immune responses and participates in the expression of various immune-related factors [57]. In crustaceans, the activation and upregulation of *LITAF* were associated with antibacterial and antiviral immune responses. Previous studies have demonstrated that, following *V. alginolyticus* infection, the expression of *LITAF* in the gill, intestine, and hepatopancreas of *L. vannamei* was significantly upregulated [58]. Additionally, after *V. parahaemolyticus* infection, the expression of *LITAF* was significantly elevated in *L. vannamei* [59]. In this study, *LITAF* was annotated in the lysosome (K19363) signaling pathway. The lysosome pathway integrates various metabolic tasks, including degradation, transport, phagocytosis, and autophagy, to maintain cellular homeostasis [60]. The upregulation of *LITAF* expression in the gill of *H. tientsinensis* is suggested to activate its innate immune response. It plays a significant role in regulating inflammatory responses and in antibacterial and antiviral immune defense mechanisms.

### 4.2. Oxidative Stress-Related Genes

Previous studies have shown that *Vibrio* infection leads to the dysregulation of the antioxidant system in crustaceans [61], resulting in oxidative stress. Oxidative stress is characterized by an imbalance between oxidation and antioxidation within the organism. Excessive oxidative stress can induce or suppress the expression of related factors, leading to inflammatory responses and causing oxidative damage.

In the experimental group of *H. tientsinensis*, the gene expression levels of Superoxide dismutase (*SOD*) and Catalase (*CAT*) were significantly upregulated in gills. SOD and CAT are key components of the enzymatic antioxidant system and are often selected as indicators of oxidative stress [62,63]. Both SOD and CAT participate in the innate immune response of invertebrates. Specifically, SOD catalyzes the conversion of O_2_ to H_2_O_2_ in response to bacterial challenge [64,65]. Conversely, CAT decomposes H_2_O_2_ into harmless H_2_O and O_2_ [66]. Following *V. parahaemolyticus* infection, the gene expression level of *SOD* in the hepatopancreas of *E. sinensis* was significantly increased [20]. Differences in the expression of non-specific immune-related enzymes, such as *AKP* and *ACP*, indicate an imbalance in the antioxidant system in crustaceans [20,37]. The significant upregulation of *SOD* and *CAT* expression, along with the notable increase in *ACP* expression in the gill of *H. tientsinensis*, suggests that *V. parahaemolyticus* infection induces oxidative stress in the gill tissue, leading to oxidative damage.

### 4.3. Genes Related to Apoptosis and Pyroptosis

Caspases are a family of proteases that participate in various critical biological processes, including apoptosis and inflammation [67]. In this study, necrosis and blurred cellular structures were observed in the gill of *H. tientsinensis* in the experimental group (Figure 1). Furthermore, there was a significant upregulation of Caspase-like (*Caspase 3*) gene expression in the gill. Caspase 3 is a conserved cysteine protease family that plays a pivotal role in apoptosis. Activated caspase 3 cleaves a series of proteins, ultimately leading to cell apoptosis [68,69]. After infection, a significant upregulation of apoptotic genes was observed in the gill.

Compared to the control group, the gene expression levels of the *IAP 1* and baculoviral *IAP* repeat-containing protein 7-like gene were significantly downregulated in the hepatopancreas of the experimental group in *H. tientsinensis*. Inhibitors of apoptosis proteins (IAPs) regulate the balance between cell proliferation and cell death by inhibiting caspase activities and mediating immune responses [70,71]. In *E. sinensis*, following LPS and bacterial stimulation, *IAP* expression increases in hemocytes, activating Caspase 3/7 and inducing apoptotic pathways [72]. IAP activity was inhibited in the hepatopancreas of *H. tientsinensis*, whereas *Caspase 3/7* exhibited no significant differential expression. This suggested that after *V. parahaemolyticus* infection in *H. tientsinensis*, the apoptotic pathways were activated, while the anti-apoptotic pathways were suppressed in the hepatopancreas.

In the hepatopancreas of the experimental group of *H. tientsinensis*, the expression of *CASP1* was significantly downregulated. The expression of *CASP1* induces pyroptosis, a form of cell death that promotes pathogen clearance by recruiting immune cells to the site of infection [73,74,75,76]. After infection, the gene expression of *CASP1* was significantly reduced in the hepatopancreas of the experimental group. Simultaneously, a significant upregulation of *Cytochrome c* expression was observed in the hepatopancreas. The release of cytochrome c from mitochondria is a hallmark of apoptosis [77]. As a caspase activator, cytochrome c induces caspase expression and activates apoptosis [78], which is consistent with the phenomenon of blurred tissue structure found in our study of hepatopancreas tissue section. After the host is attacked by *V. parahaemolyticus*, the epithelial cells atrophy and the nuclei collapse. These findings suggest that after *V. parahaemolyticus* infection, the hepatopancreas of *H. tientsinensis* experiences tissue damage, with anti-apoptotic mechanisms being suppressed and apoptotic genes being highly expressed.

## 5. Conclusions

**Figure 5:**
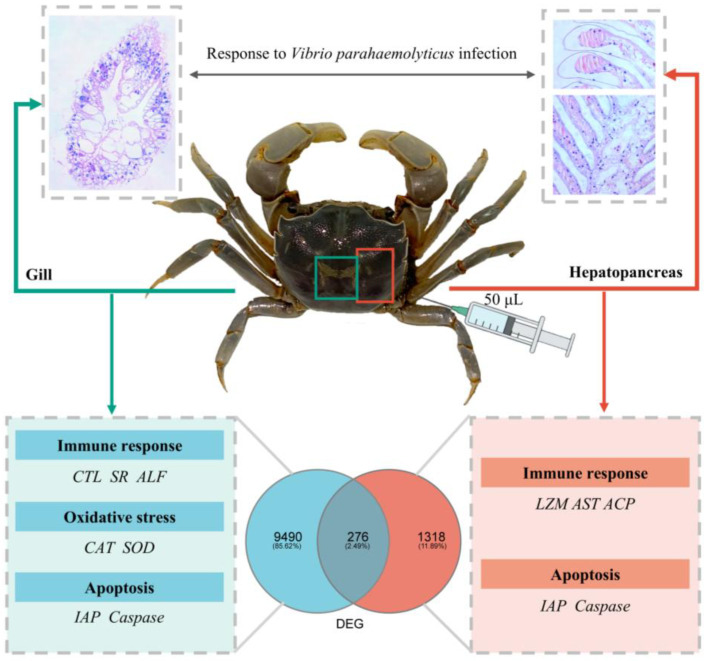
The molecular mechanism of *H. tientsinensis* gills and hepatopancreas response to *V. parahaemolyticus* infection.

The results of this study indicate that *V. parahaemolyticus* infection causes severe tissue damage in the gill and hepatopancreas of *H. tientsinensis*. In the gill, this is manifested as the proliferation and aggregation of hemocytes, with some gill filaments showing signs of breakage. In the hepatopancreas, vacuolization, basal membrane separation, and cell membrane rupture were observed. *V. parahaemolyticus* infection leads to the differential expression of immune-related genes (including *AST*, *ALT*, *LZM*, *ALF*, *LITAF*, etc.) in the gills and hepatopancreas, thereby triggering immune defense mechanisms (Table 2 and Table 3). Moreover, the expression levels of antioxidant enzymes in the gill were increased, including a rise in the activity of the non-specific immune enzyme *ACP* and a decrease in *AKP* activity, suggesting an imbalance in antioxidant system activity. Furthermore, the significant downregulation of the anti-apoptotic gene *IAP* and the significant upregulation of apoptotic genes, including *Caspase-like* and *Cytochrome c*, indicate that cellular programmed death caused by exogenous invaders leads to tissue damage in the gill and hepatopancreas of *H. tientsinensis*. In summary, we demonstrated the response mechanism of the gills and hepatopancreas of *H. tientsinensis* under *V. parahaemolyticus* infection (Figure 5). This study provides both morphological and bioinformatics data to further understand the innate immune defense mechanisms of *H. tientsinensis* against *Vibrio* species and offers theoretical insights for large-scale aquaculture practices.

## Figures and Tables

**Table 1:** General information of the transcriptome from healthy crab.

Database	Exp_Unigene Number (Percent)	Exp_Transcript Number (Precent)	All_Unigene Number (Precent)	All_Transcript Number (Precent)
GO	20,666 (22.03%)	31,379 (21.74%)	20,962 (21.86%)	31,777 (21.61%)
KEGG	19,386 (20.67%)	30,061 (20.83%)	19,668 (20.51%)	30,445 (20.71%)
eggNOG	23,773 (25.35%)	37,263 (25.82%)	24,074 (25.11%)	37,679 (25.63%)
NR	36,182 (38.58%)	58,368 (40.45%)	36,689 (38.27%)	59,102 (40.2%)
Swiss-Prot	19,161 (20.43%)	30,194 (20.92%)	19,375 (20.21%)	30,501 (20.75%)
Pfam	20,620 (21.99%)	33,750 (23.39%)	20,793 (21.69%)	34,030 (23.15%)
Total_anno	39,169 (41.76%)	62,414 (43.25%)	39,743 (41.45%)	63,229 (43.01%)
Total	93,791 (100%)	144,311 (100%)	95,878 (100%)	147,021 (100%)

**Table 2:** DEGs in the gill.

Immune Gene	NR Description	Regulation	log_2_FC
*XP_050707313.1*	baculoviral IAP repeat-containing protein 7-like	up	2.11004846858
*XP_050713859.1*	caspase-like	up	1.21144456479
*XP_055388818.1*	peroxisomal catalase-like	up	2.29787720409
*AAP93637.2*	Cu/Zn superoxide dismutase	up	2.39786254809
*XP_033114365.1*	superoxide dismutase [Mn], mitochondrial-like	up	3.26009252810
*XP_043643659.1*	lipopolysaccharide-induced tumor necrosis factor-alpha factor homolog	up	1.85199931240
*XP_050686027.1*	C-type lectin domain family 6 member A-like	up	1.45539557671
*XP_052761447.1*	scavenger receptor class F member 1-like	up	2.80249497366
*XP_050689758.1*	acid phosphatase type 7-like	up	5.54549241063
*CAI8058558.1*	Probable GH family 25 lysozyme 2	up	1.80022048962
*XP_050727498.1*	anti-lipopolysaccharide factor-like	up	7.21866970762
*XP_050727500.1*	anti-lipopolysaccharide factor-like	up	6.53449003998
*XP_026462568.1*	alanine aminotransferase 1-like	up	1.75217166318
*XP_034337692.1*	aspartate aminotransferase, mitochondrial	up	2.89772210593
*XP_014247769.1*	aspartate aminotransferase, mitochondrial	up	1.57787836676

**Table 3:** DEGs in the hepatopancreas.

Immune Gene	NR Description	Regulation	log_2_FC
*WAS29203.1*	inhibitor of apoptosis protein 1	down	2.87308466464
*XP_050738051.1*	caspase-1-like	down	4.26808751308
*XP_045105030.1*	alkaline phosphatase-like	down	2.79366226575
*XP_050689758.1*	acid phosphatase type 7-like	down	6.61045995700
*XP_050716204.1*	lysozyme-like	down	1.31142731712
*XP_050686298.1*	baculoviral IAP repeat-containing protein 7-like	down	6.36488561800
*XP_050729782.1*	aspartate aminotransferase-like	up	1.33082013560

## Data Availability

The data presented in this study are available on request from the corresponding author. The raw sequence data had been submitted to the NCBI BioSample Database with accession numbers from SAMN50293426 to SAMN50293437. Due to the confidentiality requirements of subsequent research, the public release date was set to 8 January 2027.

## Supplementary Materials

The following supporting information can be downloaded at: https://www.mdpi.com/article/10.3390/ani15192855/s1, Figure S1: PCA score plots of *H. tientsinensis* gills and hepatopancreas in the PBS control group and *V. parahaemolyticus* infection group; Figure S2: The annotation results of the GO (a) and KEGG (b) functions of unigenes; Table S1: Primer sequences used in this study; Table S2: Assembly statistic of transcriptome sequencing in *H. tientsinensis*; Table S3: Summary of sample sequencing data quality.

## References

[1] Zhang D., Ding G., Zhang H., Tang B. (2009). Isolation and characterization of 10 microsatellite markers in *Helice tientsinensis* (Brachyura: Varunidae). Conserv. Genet. Resour..

[2] Zhang L., Lan S., Angelini C., Yi H., Zhao L., Chen L., Han G. (2021). Interactive effects of crab herbivory and spring drought on a *Phragmites australis*-dominated salt marsh in the Yellow River Delta. Sci. Total Environ..

[3] Qiu D., Yan J., Ma X., Luo M., Wang Q., Cui B. (2019). Microtopographical modification by a herbivore facilitates the growth of a coastal saltmarsh plant. Mar. Pollut. Bull..

[4] Song Y., Yan C., Gao C., Xu H., Hua E., Liu X. (2022). Seasonal Distribution of Meiofaunal Assemblages in the Mangrove Tidal Flat of Futian, Shenzhen, China. J. Ocean Univ. China.

[5] Chen Z., Li B., Zhong Y., Chen J. (2004). Local competitive effects of introduced *Spartina alterniflora* on *Scirpus mariqueter* at Dongtan of Chongming Island, the Yangtze River estuary and their potential ecological consequences. Hydrobiologia.

[6] Elmer W.H. (2014). A Tripartite Interaction Between *Spartina alterniflora*, *Fusarium palustre*, and the Purple Marsh Crab (*Sesarma reticulatum*) Contributes to Sudden Vegetation Dieback of Salt Marshes in New England. Phytopathology.

[7] Lan S.Q., Zhang L.W., Yi H.P., Xu C.L., Lu F., Feng G.H., Han G.X. (2020). Food source and feeding habit of *Helice tientsinensis* from the common reed vegetation in high marsh of Yellow River Delta, China. Chin. J. Appl. Ecol..

[8] Reinsel K.A. (2004). Impact of fiddler crab foraging and tidal inundation on an intertidal sandflat: Season-dependent effects in one tidal cycle. J. Exp. Mar. Biol. Ecol..

[9] Webb A.P., Eyre B.D. (2004). The effect of natural populations of the burrowing and grazing soldier crab (*Mictyris longicarpus*) on sediment irrigation, benthic metabolism and nitrogen fluxes. J. Exp. Mar. Biol. Ecol..

[10] Meziane T., Dagata F., Lee S. (2006). Fate of mangrove organic matter along a subtropical estuary: Small-scale exportation and contribution to the food of crab communtities. Mar. Ecol. Prog Ser..

[11] Li D., Zhang J., Chen L., Lloyd H., Zhang Z.W. (2020). Burrow ambient temperature influences *Helice* crab activity and availability for migratory Red-crowned cranes *Grus japonensis*. Ecol. Evol..

[12] Huang Z.S., Aweya J.J., Zhu C.H., Tran N.T., Hong Y.J., Li S.K., Yao D.F., Zhang Y.L. (2020). Modulation of crustacean innate immune response by amino acids and their metabolites: Inferences from other species. Front. Immunol..

[13] Fajardo C., Martinez-Rodriguez G., Costas B., Mancera J.M., Fernandez-Boo S., Rodulfo H., De Donato M. (2022). Shrimp immune response: A transcriptomic perspective. Rev. Aquacult..

[14] Goulden E.F., Hall M.R., Bourne D.G., Pereg L.L., Hoj L. (2012). Pathogenicity and Infection Cycle of *Vibrio owensii* in larviculture of the Ornate Spiny Lobster (*Panulirus ornatus*). Appl. Environ. Microbiol..

[15] Lafferty K.D., Harvell C.D., Conrad J.M., Friedman C.S., Kent M.L., Kuris A.M., Powell E.N., Rondeau D., Saksida S.M. (2015). Infectious Diseases Affect Marine Fisheries and Aquaculture Economics. Annu. Rev. Mar. Sci..

[16] Ma S., Kim A., Lee W., Kim S., Lee S., Yoon D., Bae J.-S., Park C.-I., Kim S. (2020). *Vibrio harveyi* Infection Significantly Alters Amino Acid and Carbohydrate Metabolism in Whiteleg Shrimp, *Litopenaeus vannamei*. Metabolites.

[17] Zhang X., Sun J.F., Han Z.R., Chen F., Lv A.J., Hu X.C., Sun X.L., Qi H.L., Guo Y.J. (2021). *Vibrio parahaemolyticus* alters the community composition and function of intestinal microbiota in Pacific white shrimp, *Penaeus vannamei*. Aquaculture.

[18] Han J.E., Tang K.F.J., Tran L.H., Lightner D.V. (2015). *Photorhabdus* insect-related (Pir) toxin-like genes in a plasmid of *Vibrio parahaemolyticus*, the causative agent of acute hepatopancreatic necrosis disease (AHPND) of shrimp. Dis. Aquat. Org..

[19] Jiao L.F., Dai T.M., Zhong S.Q., Jin M., Sun P., Zhou Q.C. (2020). *Vibrio parahaemolyticus* infection impaired intestinal barrier function and nutrient absorption in *Litopenaeus vannamei*. Fish Shellfish Immunol..

[20] Chen D.D., Xin Y.T., Teng J., Zhao X.D., Lu J.B., Li Y.B., Wang H. (2024). Comparative transcriptomic and molecular biology analyses to explore potential immune responses to *Vibrio parahaemolyticus* challenge in *Eriocheir sinensis*. Front. Cell. Infect. Microbiol..

[21] Huang X.L., Feng Y., Duan H.M., Zhao L., Yang C., Geng Y., Ouyang P., Chen D.F., Yin L.Z., Yang S.Y. (2021). Evaluation of pathology and environmental variables contributing to hepatopancreatic necrosis syndrome of chinese mitten crab, *Eriocheir sinensis*. Ecotox. Environ. Saf..

[22] Liu H.R., Song C.W., Ning J.H., Liu Y., Cui Z.X. (2020). Identification, functional characterization and the potential role of variable lymphocyte receptor EsVLRA from *Eriocheir sinensis* in response to secondary challenge after *Vibrio parahaemolyticus* vaccine. Fish Shellfish Immunol..

[23] Sun D., Lv J., Li Y., Wu J., Liu P., Gao B. (2023). Comparative Transcriptome Analysis of the Response to *Vibrio parahaemolyticus* and Low-Salinity Stress in the Swimming Crab *Portunus trituberculatus*. Biology.

[24] Cheng C.H., Liu X.Z., Ma H.L., Liu G.X., Deng Y.Q., Feng J., Jie Y.K., Guo Z.X. (2021). The role of caspase 3 in the mud crab (*Scylla paramamosain*) after *Vibrio parahaemolyticus* infection. Fish Shellfish Immunol..

[25] Letchumanan V., Yin W.F., Lee L.H., Chan K.G. (2015). Prevalence and antimicrobial susceptibility of *Vibrio parahaemolyticus* isolated from retail shrimps in Malaysia. Front. Microbiol..

[26] Lopez-Joven C., de Blas I., Furones M.D., Roque A. (2015). Prevalences of pathogenic and non-pathogenic *Vibrio parahaemolyticus* in mollusks from the Spanish Mediterranean Coast. Front. Microbiol..

[27] Huang P., Cao L.P., Du J.L., Gao J.C., Zhang Y.N., Sun Y., Li Q.J., Nie Z.J., Xu G.H. (2023). Effects of Prometryn Exposure on Hepatopancreas Oxidative Stress and Intestinal Flora in *Eriocheir sinensis* (Crustacea: Decapoda). Antioxidants.

[28] Cervellione F., McGurk C., Silva P., Owen M.A.G., Van den Broeck W. (2016). Optimization of fixation methods for image analysis of the hepatopancreas in whiteleg shrimp, *Penaeus vannamei* (Boone). J. Fish Dis..

[29] Ching T., Huang S., Garmire L.X. (2014). Power analysis and sample size estimation for RNA-Seq differential expression. RNA.

[30] Chen L., Hua Y., Ji W., Wang J., Zhao H., Wang Z. (2023). Cloning, characterization, and expression analysis of the *CHITINASE* gene family in *Helice tientsinensis*. PeerJ.

[31] Persch T.S.P., Weimer R.N., Freitas B.S., Oliveira G.T. (2017). Metabolic parameters and oxidative balance in juvenile *Rhamdia quelen* exposed to rice paddy herbicides: Roundup, primoleo, and facet. Chemosphere.

[32] Henry R.P., Lucu C., Onken H., Weihrauch D. (2012). Multiple functions of the crustacean gill: Osmotic/ionic regulation, acid-base balance, ammonia excretion, and bioaccumulation of toxic metals. Front. Physiol..

[33] Tang D., Shi X.L., Guo H.Y., Bai Y.Z., Shen C.C., Zhang Y.P., Wang Z.F. (2020). Comparative transcriptome analysis of the gills of *Procambarus clarkii* provides novel insights into the immune-related mechanism of copper stress tolerance. Fish Shellfish Immunol..

[34] Lin Y., Huang J.J., Dahms H.U., Zhen J.J., Ying X.P. (2017). Cell damage and apoptosis in the hepatopancreas of *Eriocheir sinensis* induced by cadmium. Aquat. Toxicol..

[35] Zhang Y., Li Z.Y., Kholodkevich S., Sharov A., Feng Y.J., Ren N.Q., Sun K. (2019). Cadmium-induced oxidative stress, histopathology, and transcriptome changes in the hepatopancreas of freshwater crayfish (*Procambarus clarkii*). Sci. Total Environ..

[36] Cheng C.H., Ma H.L., Deng Y.Q., Feng J., Jie Y.K., Guo Z.X. (2020). Effects of *Vibrio parahaemolyticus* infection on physiological response, histopathology and transcriptome changes in the mud crab (*Scylla paramamosain*). Fish Shellfish Immunol..

[37] Dai C.J., Xiao L., Mo A.J., Yuan Y.C., Yuan J.F., Gu Z.M., Wang J.H. (2023). Effect of dietary bacillus subtilis supplement on cd toxicokinetics and cd-induced immune and antioxidant impairment of *Procambarus clarkii*. Environ. Sci. Pollut. Res..

[38] Fu L.L., Zhou G., Pan J.L., Li Y.H., Lu Q.P., Zhou J., Li X.G. (2017). Effects of *Astragalus* polysaccharides on antioxidant abilities and non-specific immune responses of chinese mitten crab, *Eriocheir sinensis*. Aquacult. Int..

[39] Peters C.B., Urich K., Pollwein R., Grzeschik K.H., Sippel A. (1989). The human lysozyme gene. Sequence organization and chromosomal localization. Eur. J. Biochem..

[40] Tassanakajon A., Somboonwiwat K., Supungul P., Tang S. (2013). Discovery of immune molecules and their crucial functions in shrimp immunity. Fish Shellfish Immunol..

[41] Liu H.P., Jiravanichpaisal P., Soderhall I., Cerenius L., Soderhall K. (2006). Antilipopolysaccharide Factor Interferes with White Spot Syndrome Virus Replication In Vitro and In Vivo in the Crayfish *Pacifastacus leniusculus*. J. Virol..

[42] Sun B., Wang Z., Zhu F. (2017). The crustin-like peptide plays opposite role in shrimp immune response to *Vibrio alginolyticus* and white spot syndrome virus (WSSV) infection. Fish Shellfish Immunol..

[43] Hamilton A., Harrington D., Sutcliffe I.C. (2000). Characterization of Acid Phosphatase Activities in the Equine Pathogen *Streptococcus equi*. Syst. Appl. Microbiol..

[44] Huang H.T., Hu Y.F., Lee B.H., Huang C.Y., Lin Y.R., Huang S.N., Chen Y.Y., Chang J.J., Nan F.H. (2022). Dietary of *Lactobacillus paracasei* and *Bifidobacterium longum* improve nonspecific immune responses, growth performance, and resistance against *Vibrio parahaemolyticus* in *Penaeus vannamei*. Fish Shellfish Immunol..

[45] Guan T.Y., Feng J.B., Zhu Q.Q., Wang L., Xie P., Wang H., Li J.L. (2023). Effects of abamectin on nonspecific immunity, antioxidation, and apoptosis in red swamp crayfish (*Procambarus clarkii*). Fish Shellfish Immunol..

[46] Stara A., Zuskova E., Kouba A., Velisek J. (2016). Effects of terbuthylazine-desethyl, a terbuthylazine degradation product, on red swamp crayfish (*Procambarus clarkii*). Sci. Total Environ..

[47] Wei K., Yang J. (2015). Oxidative damage of hepatopancreas induced by pollution depresses humoral immunity response in the freshwater crayfish *Procambarus clarkii*. Fish Shellfish Immunol..

[48] Wang C., Li P.F., Hu D.G., Wang H. (2023). Effect of *Clostridium butyricum* on intestinal microbiota and resistance to *Vibrio alginolyticus* of *Penaeus vannamei*. Fish Shellfish Immunol..

[49] Gough P.J., Siamon G. (2000). The role of scavenger receptors in the innate immune system. Microbes. Infect..

[50] Silverstein R.L. (2009). Inflammation, atherosclerosis, and arterial thrombosis: Role of the scavenger receptor CD36. Clev. Clin. J. Med..

[51] Rahaman S.O., Zhou G., Silverstein R.L. (2011). Vav protein guanine nucleotide exchange factor regulates CD36 protein-mediated macrophage foam cell formation via calcium and dynamin-dependent processes. J. Biol. Chem..

[52] Dambuza I.M., Brown G.D. (2015). C-type lectins in immunity: Recent developments. Curr. Opin. Immunol..

[53] Tang M.X., Li X.J., Yang L., Wang Q., Li W.W. (2020). A class B scavenger receptor mediates antimicrobial peptide secretion and phagocytosis in Chinese mitten crab (*Eriocheir sinensis*). Dev. Comp. Immunol..

[54] Wu Y.M., Yang L., Li X.J., Li L., Wang Q., Li W.W. (2017). A class B scavenger receptor from *Eriocheir sinensis* (*Es*SR-B1) restricts bacteria proliferation by promoting phagocytosis. Fish Shellfish Immunol..

[55] Huang Y., Huang X., Wang Z., Tan J.M., Hui K.M., Wang W., Ren Q. (2014). Function of two novel single-CRD containing C-type lectins in innate immunity from *Eriocheir sinensis*. Fish Shellfish Immunol..

[56] Li S., Jia Z.R., Li X.J., Geng X.Y., Sun J.S. (2014). Identification and expression analysis of lipopolysaccharide-induced TNF-alpha factor gene in Chinese mitten crab *Eriocheir sinensis*. Fish Shellfish Immunol..

[57] Yang D., Wei X., Yang J., Yang J., Xu J., Fang J., Wang S., Liu X. (2013). Identification of a LPS-induced tnf-α factor (LITAF) from mollusk *Solen grandis* and its expression pattern towards PAMPS stimulation. Fish Shellfish Immunol..

[58] Wang P.H., Wan D.H., Pang L.R., Gu Z.H., Qiu W., Weng S.P., Yu X.Q., He J.G. (2012). Molecular cloning, characterization and expression analysis of the tumor necrosis factor (TNF) superfamily gene, TNF receptor superfamily gene and lipopolysaccharide-induced tnf-α factor (LITAF) gene from *Litopenaeus vannamei*. Dev. Comp. Immunol..

[59] Jiao L., Dai T., Zhong S., Jin M., Sun P., Zhou Q. (2020). *Vibrio parahaemolyticus* Infection Influenced Trace Element Homeostasis, Impaired Antioxidant Function, and Induced Inflammation Response in *Litopenaeus vannamei*. Biol. Trace Elem. Res..

[60] Wu Y., Wang H., Xu H. (2025). Autophagy-lysosome pathway in insulin & glucagon homeostasis. Front. Endocrinol..

[61] Subash P., Chrisolite B., Sivasankar P., Rosalind George M., Vijay Amirtharaj K.S., Padmavathy P., Rani V., Sankar Sri Balaje R., Gowtham S., Mageshkumar P. (2023). White feces syndrome in *Penaeus vannamei* is potentially an *Enterocytozoon hepatopenaei* (EHP) associated pathobiome origin of *Vibrio* spp. *J*. Invertebr. Pathol..

[62] Ighodaro O.M., Akinloye O.A. (2019). First line defence antioxidants-superoxide dismutase (SOD), catalase (CAT) and glutathione peroxidase (GPX): Their fundamental role in the entire antioxidant defence grid. Alex. J. Med..

[63] Wang H., Li E., Huang Q., Liu J., Miao Y., Wang X., Qin C., Qin J., Chen L., Xu H. (2024). Growth and Hepatopancreas Health of Juvenile Chinese Mitten Crab (*Eriocheir sinensis*) Fed Different Levels of Black Soldier Fly (*Hermetia illucens*) Larvae Meal for Fish Meal Replacement. Aquac. Nutr..

[64] Hung M.N., Shiomi R., Nozaki R., Kondo H., Hirono I. (2014). Identification of novel copper/zinc superoxide dismutase (Cu/ZnSOD) genes in kuruma shrimp *Marsupenaeus japonicus*. Fish Shellfish Immunol..

[65] Qu R.J., Feng M.B., Sun P., Wang Z.Y. (2015). A Comparative Study on Antioxidant Status Combined with Integrated Biomarker Response in *Carassius auratus* Fish Exposed to Nine Phthalates. Environ. Toxicol..

[66] Atli G., Canli E.G., Eroglu A., Canli M. (2016). Characterization of antioxidant system parameters in four freshwater fish species. Ecotox. Environ. Saf..

[67] Ren X.Y., Yu X., Gao B.Q., Liu P., Li J. (2017). Characterization of three caspases and their pathogen-induced expression pattern in *Portunus trituberculatus*. Fish Shellfish Immunol..

[68] Boatright K.M., Salvesen G.S. (2003). Mechanisms of caspase activation. Curr. Opin. Cell Biol..

[69] Reis M.I., Nascimento D.S., do Vale A., Silva M.T., dos Santos N.M. (2007). Molecular cloning and characterisation of sea bass (*Dicentrarchus labrax* L.) caspase-3 gene. Mol. Immunol..

[70] Hermann S. (1995). Mechanisms and Genes of Cellular Suicide. Science.

[71] Kiss T. (2010). Apoptosis and its functional significance in molluscs. Apoptosis.

[72] Qu C., Sun J.J., Xu Q.S., Lv X.J., Yang W., Wang F.F., Wang Y., Yi Q.L., Jia Z.H., Wang L.L. (2019). An inhibitor of apoptosis protein (*Es*IAP1) from Chinese mitten crab *Eriocheir sinensis* regulates apoptosis through inhibiting the activity of *Es*caspase-3/7-1. Sci. Rep..

[73] Miao E.A., Alpuche-Aranda C.M., Dors M., Clark A.E., Bader M.W., Miller S.I., Aderem A. (2006). Cytoplasmic flagellin activates caspase-1 and secretion of interleukin 1β via Ipaf. Nat. Immunol..

[74] Wang Y.P., Gao W.Q., Shi X.Y., Ding J.J., Liu W., He H.B., Wang K., Shao F. (2017). Chemotherapy drugs induce pyroptosis through caspase-3 cleavage of a Gasdermin. Nature.

[75] Yang G., Wang J.J., Luo T., Zhang X.B. (2019). White spot syndrome virus infection activates Caspase 1-mediated cell death in crustacean. Virology.

[76] Yazdi A.S., Guarda G., Riteau N., Drexler S.K., Tardivel A., Couillin I., Tschopp J. (2010). Nanoparticles activate the NLR pyrin domain containing 3 (Nlrp3) inflammasome and cause pulmonary inflammation through release of IL-1α and IL-1β. Proc. Natl. Acad. Sci. USA.

[77] Bertheloot D., Latz E., Franklin B.S. (2021). Necroptosis, pyroptosis and apoptosis: An intricate game of cell death. Cell. Mol. Immunol..

[78] Kalkavan H., Chen M.J., Crawford J.C., Quarato G., Fitzgerald P., Tait S.W.G., Goding C.R., Green D.R. (2022). Sublethal cytochrome c release generates drug-tolerant persister cells. Cell.

