# Supplementary material for: Transcriptome Analysis of Potential Genes Involved in Innate Immunity in Mudflat Crab (Helice tientsinensis)
Source: Animals (Basel). 2025 Sep 30;15(19):2855. doi: 10.3390/ani15192855 (PMC12524317; doi:10.3390/ani15192855)
Supplement: Supplementary file 1 [file animals-15-02855-s001.zip › Figure S1 PCA score plots of H. tientsinensis gills and hepatopancreas in the PBS control group and V. parahaemolyticus infection group.pdf]

Supplementary Materials of Transcriptome analysis of potential genes involved in innate immunity in mudflat crab (*Helice tientsinensis*)

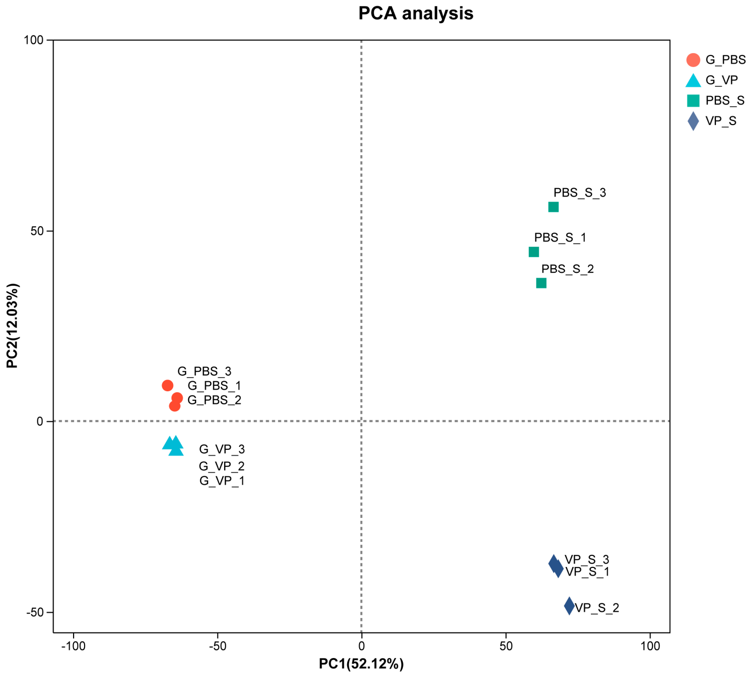

Figure S1 PCA score plots of *H. tientsinensis* gills and hepatopancreas in the PBS control group and *V. parahaemolyticus* infection group. G_PBS: The PBS group of gills. G_VP: The VP group of gills. PBS_S: The PBS group of hepatopancreas. VP_S: The PBS group of hepatopancreas.
