# Supplementary material for: Transcriptome Analysis of Potential Genes Involved in Innate Immunity in Mudflat Crab (Helice tientsinensis)
Source: Animals (Basel). 2025 Sep 30;15(19):2855. doi: 10.3390/ani15192855 (PMC12524317; doi:10.3390/ani15192855)
Supplement: Supplementary file 1 [file animals-15-02855-s001.zip › Figure S2 The annotation results of the GO (a) and KEGG (b) functions of unigenes.pdf]

Supplementary Materials of Transcriptome analysis of potential genes involved in innate immunity in mudflat crab (*Helice tientsinensis*)

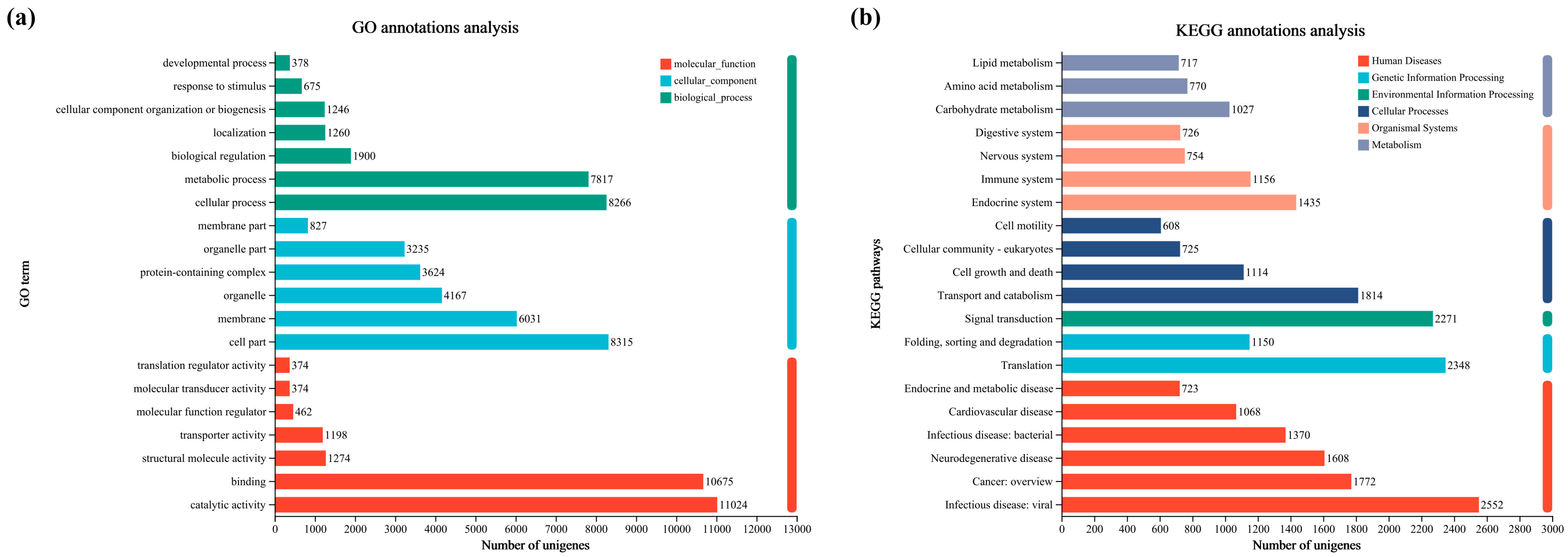

Figure S2 The annotation results of the GO (a) and KEGG (b) functions of unigenes.
