# Supplementary material for: Transcriptome Analysis of Potential Genes Involved in Innate Immunity in Mudflat Crab (Helice tientsinensis)
Source: Animals (Basel). 2025 Sep 30;15(19):2855. doi: 10.3390/ani15192855 (PMC12524317; doi:10.3390/ani15192855)
Supplement: Supplementary file 1 [file animals-15-02855-s001.zip › Table S1 Primer sequences used in this study.pdf]

Supplementary Materials of Transcriptome analysis of potential genes involved in innate immunity in mudflat crab (*Helice tientsinensis*)

Table S1 Primer sequences used in this study

Gene description	Forward primer sequence (5′-3′)	Reverse primer sequence (5′-3′)
PEXCAT	GCCCTGTTGTCATTTCCGACTTCCA	GGTTGGAGTGACCTTACCGATGGAT
ACP7	GGATGAAGCCGCTCTCAAGGCAATA	TACAGTCGTCGTGGTCGTTGTTGGA
ALF	CTTTGTTCATCACCCTGGTCCTGC	TTTCAGTGGGCGAAGACATTAGG
IAP1	GTGTGGCTTTTATGATACTTAGGTCC	GTGGTGCCCAGAGTGTAACTATGT
CASP1	CAGGACCGCCACTCCCATCCATCA	ACGAAGTATCCGTTGGGTCTTTTGTGG
AKP	GGGATGTAGGCGTTCTCGTGGATGC	CTGGGACTGGCGGACTACTCTGACG
GAPDH	GTCTCCAATGCCTCCTGC	GCACTCCTTGCCTAAGATAACA
