# Supplementary material for: Transcriptome Analysis of Potential Genes Involved in Innate Immunity in Mudflat Crab (Helice tientsinensis)
Source: Animals (Basel). 2025 Sep 30;15(19):2855. doi: 10.3390/ani15192855 (PMC12524317; doi:10.3390/ani15192855)
Supplement: Supplementary file 1 [file animals-15-02855-s001.zip › Table S2 Assembly statistic of transcriptome sequencing in H. tientsinensis.pdf]

Supplementary Materials of Transcriptome analysis of potential genes involved in innate immunity in mudflat crab (*Helice tientsinensis*)

Table S2 Assembly statistic of transcriptome sequencing in *H. tientsinensis*

Sample	Raw reads	Raw bases	Clean reads	Clean bases	Error rate (%)	Q20(%)	Q30(%)	GC content (%)
H_PBS_1	44306350	6690258850	43563120	6501668839	0.0242	98.34	94.96	51.03
H_PBS_2	43644338	6590295038	42716384	6390313149	0.0241	98.4	95.12	50.54
H_PBS_3	46799920	7066787920	46091290	6877643748	0.024	98.43	95.17	51.22
H_VP_1	51677084	7803239684	50820738	7592977535	0.0243	98.32	94.88	50.71
H_VP_2	51257412	7739869212	50853732	7618553471	0.0119	98.81	96.28	48.75
H_VP_3	45262890	6834696390	44570894	6630450370	0.0236	98.56	95.59	50.33
G_PBS_1	48041212	7254223012	47380284	7015115879	0.0248	98.12	94.45	47.98
G_PBS_2	49449642	7466895942	48723060	7243048709	0.0245	98.23	94.71	48.52
G_PBS_3	44393846	6703470746	43729652	6517114287	0.0246	98.19	94.58	47.67
G_VP_1	46152978	6969099678	45669184	6812050171	0.0244	98.25	94.77	49.61
G_VP_2	49535448	7479852648	48604150	7238199011	0.0243	98.28	94.94	49.44
G_VP_3	54513750	8231576250	53564604	7948192454	0.0243	98.28	94.88	49.07
