# Supplementary material for: Transcriptome Analysis of Potential Genes Involved in Innate Immunity in Mudflat Crab (Helice tientsinensis)
Source: Animals (Basel). 2025 Sep 30;15(19):2855. doi: 10.3390/ani15192855 (PMC12524317; doi:10.3390/ani15192855)
Supplement: Supplementary file 1 [file animals-15-02855-s001.zip › Table S3 Summary of sample sequencing data quality.pdf]

Supplementary Materials of Transcriptome analysis of potential genes involved in innate immunity in mudflat crab (*Helice tientsinensis*)

Table S3 Summary of sample sequencing data quality

Type	Unigene	Transcript
Total number	95878	147021
Total base	80870673	136639469
Fragment mapped percent(%)	71.419	83.393
GC percent (%)	45.63	46.06
TransRate score	0.34302	0.38946
